# Specific Types of Physical Exercises, Dietary Preferences, and Obesity Patterns With the Incidence of Hypertension: A 26-years Cohort Study

**DOI:** 10.3389/ijph.2021.1604441

**Published:** 2022-01-27

**Authors:** Xin Wang, Fayun Zhao, Qiang Zhao, Kun Wang, Shenke Kong, Peiyao Ma, Bingsen Huang, Changchun Du

**Affiliations:** Henan Provincial Chest Hospital, Zhengzhou, China

**Keywords:** cohort study, obesity patterns, physical exercise, dietary preferences, incident hypertension

## Abstract

**Objectives:** To examine the associations of specific types of physical exercises, dietary preferences, and obesity patterns with incident hypertension.

**Methods:** In this cohort study, obesity patterns were defined using general and abdominal obesity as G-/A-, G+/A- or G-/A+, and G+/A+. The type of physical exercises and dietary preferences were collected using a validated questionnaire. Participants with systemic blood pressure/diastolic blood pressure ≥140 mmHg/90 mmHg, use of antihypertensive medications, or a self-reported diagnosis were identified as hypertension.

**Results:** There were 10,713 participants in this study. Martial arts, gymnastics, and ping pong could decrease the risk of hypertension (*HR*: 0.792, 0.884, and 0.855; and *95% CI*: 0.743–0.845, 0.825–0.948, and 0.767–0.953, respectively). However, TV or computer usage, and consumption of fast food, soft/sugared drinks, and salty snack food could increase incident hypertension (*HR*: 1.418, 1.381, 1.233, and 1.225; and *95% CI*: 1.315–1.529, 1.269–1.504, 1.157–1.314, and 1.139–1.316, respectively). Obese subjects had an increased risk of hypertension.

**Conclusion:** The type of physical exercises, dietary preferences, and obesity patterns were associated with incident hypertension. More attention should be paid to these lifestyles to benefit health outcomes.

## Introduction

Hypertension plays a key role in the development of cardiovascular disease and cerebrovascular disease [1–3]. Therefore, hypertension has been an important public health concern worldwide. Recently, hypertensive individuals accounted for approximately 25% of the adult population, and this proportion is expected to be 29% by 2025 [4]. In China, the prevalence of hypertension has reached 23.2%, accounting for 244.5 million of the Chinese adult population [5]. Therefore, it is imperative to take active measurements to control and prevent hypertension.

It has been established that obesity, physical activity, and diet were strongly associated with the development of hypertension [6–10]. However, both general and abdominal obesity were considered as the main risk factors of hypertension. There might be an interaction between general and abdominal obesity. Therefore, obesity patterns defined by general and abdominal obesity might be more comprehensive in predicting the risk of hypertension. It is documented that physical exercise has multiple health benefits, especially for cardiovascular disease [11]. However, little is known about the associations of specific types of physical exercises with the incidence of hypertension. There were however some studies to investigate the relationship between dietary intakes and hypertension [12–14]. Given the complex climate and diversity of culture in China, dietary preferences varied across different provinces. Therefore, it was important to evaluate the associations of dietary preferences with the risk of hypertension on a national scale.

Therefore, this study was designed to explore the associations of specific types of physical exercises, dietary preferences, and obesity patterns with the incidence of hypertension. It was expected that this study would provide accurate evidence for the control and prevention of hypertension.

## Methods

### Study Design

Data analyzed in this study were obtained from the China Health and Nutrition Survey (CHNS), which was a 26-years national cohort study from 1989 to 2015 and covered nine provinces. A multistage random stratified cluster sampling method was used to select nine provinces from the 31 provinces of China to make the study representative across geography, economic development, and health indicators. Then, four counties were selected in each province using stratified random sampling by income. Villages or neighborhoods within those four counties were sampled randomly. Participants might enter into the CHNS in different waves. The main study population remained fixed between waves 1989 and 1993. Since the wave of 1997, a few new households were added to the survey to replace lost-to-follow-up participants. Meanwhile, an additional province of Heilongjiang was added. Since wave 2011, three megacities of Beijing, Chongqing, and Shanghai were added. In the CHNS, information on household survey, health and nutrition survey, physical activity, aging , body image, mass media behaviors and practices, marraige status, community survey, food market survey, and health and family planning facility surveys were collected. Especially, individual data on demographics, health-related behaviors, health history, dietary intake, blood pressure, and body composition were collected using a validated questionnaire. The details of analysis goals, survey design, and data collection in the CHNS were found in the literature published elsewhere [15].

### Study Population

All subjects entering into the CHNS before wave 2015 and aged 18 years or over at baseline were the targeted population. Participants with any of the following items would be excluded: i. hypertension at baseline, ii. missing data, and iii. implausible outlying data (e.g., a weight >300 kg or <20 kg, a waist circumference (WC) > 200 cm or <20 cm).

### Exposure Variables

Obesity patterns were defined using general and abdominal obesity, which were calculated using body mass index (BMI) and WC, respectively. BMI was calculated as weight in kilograms divided by height squared in meters, both of which were measured according to the standardized protocol. Participants with a BMI ≥28 kg/m^2^ were considered to have general obesity [16]. Otherwise, participants with a BMI <28 kg/m^2^ were identified as normal weight or overweight. Similarly, males with a WC ≥ 85 cm or females with a WC ≥ 80 cm were considered to have abdominal obesity [16]. Obesity patterns were defined as follows: G-/A- indicated participants without neither general obesity nor abdominal obesity, G+/A- or G-/A+ indicated participants with either general obesity or abdominal obesity, and G+/A+ indicated participants with both general and abdominal obesity.

The type of physical exercises in this study included martial arts, track and field, gymnastics, ping pong, badminton or volleyball, and soccer or basketball. Martial arts included Kung Fu and Tai Chi. Track and field included walking and running. And gymnastics included dancing and acrobatics. Additionally, sedentary behavior included TV or computer usage and reading or writing. Each type of physical exercise was collected using a validated questionnaire via a similar question: Do you participate in this activity? 0 indicates no, 1 indicates yes, and 9 indicates unknown [17]. In the final analysis, the option of “unknown” was removed. Since physical exercise types involved in the CHNS were limited to only those mentioned above, other physical exercises, such as cycling and swimming, were not analyzed in this study.

Similarly, each participant was asked their dietary preferences using a similar question: How much do you like this food? [18] The options included five items, which ranked in order from 1 indicating dislike very much to 5 indicating like very much. If participants did not eat this food, the number of 9 was assigned. Dietary preferences were dichotomized as follows: “dislike” included options 1, 2, and 9, and “like” included options 3, 4, and 5.

### The Definition of Hypertension

The first wave of the CHNS was conducted in 1989, and the subsequent follow-up waves were conducted in 1991, 1993, 1997, 2000, 2004, 2006, 2009, 2011, and 2015, respectively. Since participants were not all recruited in 1989, the follow-up times of all participants were different. When participants entered into the CHNS, they were interviewed for blood pressure in each wave. If participants were firstly diagnosed with hypertension in a wave, the exact time when firstly diagnosed with hypertension was further checked. Each participant was required to rest for 10 min in the seated position before measurement of blood pressure. Suitable cuff sizes were chosen according to the upper arm circumference. A standard mercury sphygmomanometer was used to measure diastolic blood pressure (DBP) and systemic blood pressure (SBP), which were indicated by the first and fifth Korotkoff sounds, respectively [19]. These measurements were repeated three times, and the averages were used in the final analysis. If participants with an SBP ≥ 140 mmHg or/and a DBP ≥ 90 mmHg, use of antihypertensive medications, or a self-reported diagnosis with hypertension, they were identified as having hypertension [19].

### Statistical Analysis

Continuous variables, such as age, BMI, and WC, were normal distribution. Therefore, means and standard deviations were employed to describe their distributions. Frequencies and constituent ratios were used to express categorical variables. Phi coefficient was used to assess the correlations across physical exercise types and food preferences. To correct the competitive risk of death, *Cox* regression with Fine-Gray competing risk model was employed to obtain *hazard ratios (HRs)* and 95% confidence intervals (*CIs*) on the associations of specific type of physical exercises, dietary preferences, and obesity patterns with the incidence of hypertension. New-onset hypertension was considered an end-event. Age-scale from birth year to the occurrence of hypertension, death, loss to follow-up, or the end of this study, whichever came first, was considered as the time variable. The proportional hazards assumption was confirmed to hold in all *Cox* regressions, and *Cox* regression was further stratified by living area. Furthermore, the interactions between sex and exposure variables on the incidence of hypertension were examined. All analyses were conducted using SAS 9.4 (SAS Institute Inc., Cary, NC, United States). A two-tailed *p* ≤ 0.05 indicated the statistical significance.

## Results

### The Baseline Characteristics

There were 10,713 participants included in the final analysis. The number of subjects with hypertension was 4,346, accounting for 40.57%. The proportions of men and women were 43.57 and 56.43%, respectively. The average age was 44.40 ± 15.42 years, and the averages of BMI and WC were 23.41 ± 3.66 kg/m^2^ and 81.54 ± 10.85 cm, respectively. (Table 1).

**TABLE 1:** The characteristics of all subjects at baseline. (Specific Types of Physical Exercises, Dietary Preferences, and Obesity Patterns With the Incidence of Hypertension: A 26-years Cohort Study, China, and 2021).

Characteristics	All subjects (10,713)	Status	
		Normal (6,367)	Hypertension (4,346)
Age (year)	44.40 ± 15.42	39.24 ± 13.79	51.96 ± 14.55
BMI (kg/m^2^)	23.41 ± 3.66	22.68 ± 3.47	24.48 ± 3.66
WC (cm)	81.54 ± 10.85	79.00 ± 10.00	85.26 ± 10.98
Sex			
Male	4,668 (43.57)	2,466 (38.73)	2,202 (50.67)
Female	6,045 (56.43)	3,901 (61.27)	2,144 (49.33)
Education degree			
Primary school or none	3,296 (30.77)	2,819 (46.12)	947 (88.01)
Middle school	6,080 (56.75)	2,898 (47.42)	118 (10.97)
College or above	1,337 (12.48)	395 (6.46)	11 (1.02)
Married status			
unmarried	788 (7.36)	648 (10.18)	140 (3.22)
married	9,128 (85.20)	5,400 (84.81)	3,728 (85.78)
Divorced/Widowed	797 (7.44)	319 (5.01)	478 (11.00)
Smoking			
No	7,634 (71.26)	4,780 (75.07)	2,854 (65.67)
Yes	3,079 (28.74)	1,587 (24.93)	1,492 (34.33)
Alcohol consumption			
No	7,099 (66.27)	4,381 (68.81)	2,718 (62.54)
Yes	3,614 (33.73)	1986 (31.19)	1,628 (37.46)
Ethnicity			
Han	9,801 (91.49)	5,728 (89.96)	4,073 (93.72)
Other	912 (8.51)	639 (10.04)	273 (6.28)
Gross family income			
Low	2,772 (25.88)	1,486 (23.34)	1,286 (29.59)
High	7,941 (74.12)	4,881 (76.66)	3,060 (70.41)
History of diabetes			
No	10,371 (96.81)	6,284 (98.70)	4,087 (94.04)
Yes	342 (3.19)	83 (1.30)	259 (5.96)
Region			
Urban	5,249 (49.00)	3,168 (49.76)	2081 (47.88)
Rural	5,464 (51.00)	3,199 (50.24)	2,265 (52.12)
Obesity patterns			
G-/A-	5,012 (46.78)	3,607 (56.65)	1,405 (32.33)
G+/A- or G-/A+	2,214 (20.67)	1,267 (19.90)	947 (21.79)
G+/A+	3,487 (32.55)	1,493 (23.45)	1994 (45.88)
Martial arts			
No	6,016 (56.16)	3,565 (55.99)	2,451 (56.40)
Yes	4,697 (43.84)	2,802 (44.01)	1895 (43.60)
Track and field			
No	10,090 (94.18)	5,979 (93.91)	4,111 (94.59)
Yes	623 (5.82)	388 (6.09)	235 (5.41)
Gymnastics			
No	7,179 (67.01)	4,080 (64.08)	3,099 (71.31)
Yes	3,534 (32.99)	2,287 (35.92)	1,247 (28.69)
Soccer or basketball			
No	7,756 (72.40)	4,356 (68.42)	3,400 (78.23)
Yes	2,957 (27.60)	2011 (31.58)	946 (21.77)
Badminton or volleyball			
No	7,733 (72.18)	4,342 (68.20)	3,391 (78.03)
Yes	2,980 (27.82)	2025 (31.80)	955 (21.97)
Ping pong			
No	9,831 (91.77)	5,892 (92.54)	3,939 (90.64)
Yes	882 (8.23)	475 (7.46)	407 (9.36)
Reading or writing			
No	6,143 (57.34)	3,610 (56.70)	2,533 (58.28)
Yes	4,570 (42.66)	2,757 (43.30)	1813 (41.72)
TV or computer usage			
No	6,893 (64.34)	3,754 (58.96)	3,139 (72.23)
Yes	3,820 (35.66)	2,613 (41.04)	1,207 (27.77)
Fast food			
No	8,058 (75.22)	4,456 (69.99)	3,602 (82.88)
Yes	2,655 (24.78)	1911 (30.01)	744 (17.12)
Soft/sugared drinks			
No	5,558 (51.88)	2,984 (46.87)	2,574 (59.23)
Yes	5,155 (48.12)	3,383 (53.13)	1772 (40.77)
Salty snack food			
No	7,306 (68.20)	4,051 (63.62)	3,255 (74.90)
Yes	3,407 (31.80)	2,316 (36.38)	1,091 (25.10)
Vegetables			
No	241 (2.25)	155 (2.43)	86 (1.98)
Yes	10,472 (97.75)	6,212 (97.57)	4,260 (98.02)
Fruits			
No	608 (5.68)	490 (9.53)	65 (11.78)
Yes	10,105 (94.32)	4,653 (90.47)	487 (88.22)

BMI: body mass index, WC: waist circumference The obesity patterns were defined as follows: G-/A- indicated participants without either general or abdominal obesity, G+/A- or G-/A+ indicated participants with either general obesity or abdominal obesity, and G+/A+ indicated participants with both general and abdominal obesity.

Correlations across physical exercise types or food preferences were performed. Supplementary Table S1 shows that there were strong correlations between martial arts and gymnastics (Phi = 0.543), between gymnastics and soccer or basketball (Phi = 0.662), and between gymnastics and badminton or volleyball (Phi = 0.658). And there was a very strong correlation between soccer or basketball and badminton or volleyball (Phi = 0.982). Supplementary Table S2 shows that a strong correlation was observed only between fast food and salty snack food (Phi = 0.583).

### The Interactions Between Sex and Exposure Variables on the Incidence of Hypertension

Table 2 shows that there were significant interactions between sex and soccer or basketball, badminton or volleyball, TV or computer usage, fast food, soft/sugared drinks, salty snack food, and vegetables (*HR:* 0.848, 0.857, 0.716, 0.756, 0.841, 0.822, and 1.847; *95% CI:* 0.701–0.996, 0.710–1.004, 0.572–0.859, 0.586–0.925, 0.715–0.966, 0.677–0.966, and 1.384–2.309; *p* = 0.029, 0.039, <0.001, 0.001, 0.007, 0.008, and 0.009, respectively).

**TABLE 2:** The interactions between sex and exposure variables on the incidence of hypertension. (Specific Types of Physical Exercises, Dietary Preferences, and Obesity Patterns With the Incidence of Hypertension: A 26-years Cohort Study, China, and 2021).

Exposure variables	*HR (95% CI)*	*p*
Martial arts	1.125 (1.002–1.247)	0.060
Track and field	1.072 (0.806–1.338)	0.609
Gymnastics	0.966 (0.833–1.098)	0.603
Soccer or basketball	0.848 (0.701–0.996)	0.029
Badminton or volleyball	0.857 (0.710–1.004)	0.039
Ping pong	1.122 (0.911–1.333)	0.286
Reading or writing	0.917 (0.788–1.046)	0.187
TV or computer usage	0.716 (0.572–0.859)	<0.001
Fast food	0.756 (0.586–0.925)	0.001
Soft/sugared drinks	0.841 (0.715–0.966)	0.007
Salty snack food	0.822 (0.677–0.966)	0.008
Vegetables	1.847 (1.384–2.309)	0.009
Fruits	0.949 (0.708–1.190)	0.670
Obesity patterns	1.052 (0.981–1.123)	0.161

### The Associations of Specific Types of Physical Exercises With the Incidence of Hypertension

Table 3 shows the associations of each specific type of physical exercise with the incidence of hypertension. In total population, martial arts, gymnastics, and ping pong were associated with a lower risk of hypertension (*HR*: 0.792, 0.884, and 0.855; and *95% CI*: 0.743–0.845, 0.825–0.948, and 0.767–0.953; and *p* < 0.001, = 0.001, and 0.005, respectively). However, TV or computer usage was associated with a higher risk of hypertension (*HR*: 1.418; and *95% CI*: 1.315–1.529, *p* < 0.001). There were no significant associations of track and field, soccer or basketball, badminton or volleyball, and reading or writing with hypertension. The results of urban areas were in line with those in the total population. However, significant associations were observed in martial arts (*p* < 0.001), track and field (*p* = 0.041), gymnastics (*p* = 0.039), and TV or computer usage (*p* < 0.001) in rural areas. The Kaplan-Meier curves of specific type of physical exercise with the incidence of hypertension are shown in Supplementary Figure S1. And the full adjustment model of the associations of the specific types of physical exercise with the incidence of hypertension is displayed in Supplementary Table S3.

**TABLE 3:** The associations of specific types of physical exercise with the incidence of hypertension. (Specific Types of Physical Exercises, Dietary Preferences, and Obesity Patterns With the Incidence of Hypertension: A 26-years Cohort Study, China, and 2021).

	HR	*HR 95% CI*	*p*
Total (N = 10,713)^a^			
Martial arts	0.792	0.743–0.845	<0.001
Track and field	0.947	0.831–1.079	0.410
Gymnastics	0.884	0.825–0.948	0.001
Soccer or basketball	0.974	0.902–1.051	0.494
Badminton or volleyball	0.970	0.898–1.047	0.430
Ping pong	0.855	0.767–0.953	0.005
Reading or writing	0.994	0.923–1.071	0.876
TV or computer usage	1.418	1.315–1.529	<0.001
Urban (N = 5,249)^b^			
Martial arts	0.782	0.714–0.857	<0.001
Track and field	1.068	0.910–1.254	0.421
Gymnastics	0.877	0.798–0.963	0.006
Soccer or basketball	0.958	0.866–1.061	0.412
Badminton or volleyball	0.953	0.861–1.055	0.355
Ping pong	0.844	0.742–0.960	0.010
Reading or writing	1.026	0.930–1.131	0.615
TV or computer usage	1.330	1.203–1.471	<0.001
Rural (N = 5,464)^b^			
Martial arts	0.809	0.739–0.885	<0.001
Track and field	0.796	0.640–0.990	0.041
Gymnastics	0.898	0.811–0.995	0.039
Soccer or basketball	1.020	0.909–1.144	0.740
Badminton or volleyball	1.017	0.907–1.141	0.770
Ping pong	0.852	0.697–1.042	0.119
Reading or writing	0.962	0.862–1.073	0.488
TV or computer usage	1.542	1.380–1.724	<0.001

a In this model, sex, smoking, alcohol consumption, ethnicity, education levels, married status, regions, gross family income, history of diabetes, and BMI, were adjusted.

b In this model, sex, smoking, alcohol consumption, ethnicity, education levels, married status, gross family income, history of diabetes, and BMI, were adjusted.

### The Associations of Dietary Preferences With the Incidence of Hypertension

The associations of dietary preferences with the risk of hypertension are displayed in Table 4. Subjects with dietary preferences of fast food, soft/sugared drinks, salty snack food, and fruit had a higher incidence of hypertension (*HR*: 1.381, 1.233, 1.225, and 1.155; and *95% CI*: 1.269–1.504, 1.157–1.314, 1.139–1.316, and 1.021–1.307, respectively). However, a dietary preference for vegetables was not associated with the development of hypertension (*p =* 0.127). When stratified by region, the results were comparable with those in the total population, except for preference for fruits in rural areas (*p =* 0.260). The Kaplan-Meier curves of dietary preferences with the incidence of hypertension are shown in Supplementary Figure S2. The full adjustment model of the associations of dietary preferences with the incidence of hypertension is displayed in Supplementary Table S4.

**TABLE 4:** The associations of dietary preferences with the incidence of hypertension. (Specific Types of Physical Exercises, Dietary Preferences, and Obesity Patterns With the Incidence of Hypertension: A 26-years Cohort Study, China, and 2021).

	HR	*HR 95% CI*	*p*
Total (N = 10,713)^a^			
Fast food	1.381	1.269–1.504	<0.001
Soft/sugared drinks	1.233	1.157–1.314	<0.001
Salty snack food	1.225	1.139–1.316	<0.001
Vegetables	1.194	0.951–1.498	0.127
Fruits	1.155	1.021–1.307	0.022
Urban (N = 5,249)^b^			
Fast food	1.220	1.076–1.383	0.002
Soft/sugared drinks	1.194	1.090–1.307	<0.001
Salty snack food	1.179	1.061–1.310	0.002
Vegetables	1.176	0.839–1.648	0.347
Fruits	1.229	1.018–1.484	0.032
Rural (N = 5,464)^b^			
Fast food	1.539	1.372–1.726	<0.001
Soft/sugared drinks	1.265	1.157–1.383	<0.001
Salty snack food	1.271	1.153–1.401	<0.001
Vegetables	1.185	0.872–1.611	0.277
Fruits	1.098	0.933–1.291	0.260

a In this model, sex, smoking, alcohol consumption, ethnicity, education levels, married status, regions, gross family income, history of diabetes, physical activity, and BMI, were adjusted.

b In this model, sex, smoking, alcohol consumption, ethnicity, education levels, married status, gross family income, history of diabetes, physical activity, and BMI, were adjusted.

### The Associations of Obesity Patterns With the Incidence of Hypertension

Compared to G-/A-, G+/A- or G-/A+, and G+/A+ could increase the incidence of hypertension. Furthermore, the effect of G+/A+ on hypertension was stronger than G+/A- or G-/A+ (*HR*: 1.803 vs 1.290; and *95% CI*: 1.624–2.003 vs. 1.141–1.459) in the total population, as shown in Table 5. In both rural and urban areas, G+/A- or G-/A+ and G+/A+ were associated with a higher risk of hypertension. Similarly, subjects with G+/A+ had a higher risk of hypertension than those with G+/A- or G-/A+. The Kaplan-Meier curve of obesity patterns with the incidence of hypertension is shown in Supplementary Figure S2. On the other hand, when obesity patterns were divided into four categories, G+/A-, G-/A+, and G+/A+ were linked to a higher incidence of hypertension (*HR*: 1.505, 1.100, and 1.768; and *95% CI*: 1.306–1.734, 1.001–1.208, and 1.646–1.898, respectively) compared to G-/A-. And the full adjustment model of the associations of obesity patterns with the incidence of hypertension is displayed in Supplementary Table S4.

**TABLE 5:** The associations of obesity patterns with the incidence of hypertension. (Specific Types of Physical Exercises, Dietary Preferences, and Obesity Patterns With the Incidence of Hypertension: A 26-years Cohort Study, China, and 2021).

	HR	*HR 95% CI*	*p*
Total (N = 10,713)^a^			
G-/A-	1	1	1
G+/A- or G-/A+	1.290	1.141–1.459	<0.001
G+/A+	1.803	1.624–2.003	<0.001
Urban (N = 5,249)^b^			
G-/A-	1	1	1
G+/A- or G-/A+	1.290	1.141–1.459	<0.001
G+/A+	1.803	1.624–2.003	<0.001
Rural (N = 5,464)^b^			
G-/A-	1	1	1
G+/A- or G-/A+	1.156	1.029–1.300	0.015
G+/A+	1.685	1.528–1.858	<0.001

a In this model, age, sex, smoking, alcohol consumption, ethnicity, education levels, married status, regions, gross family income, history of diabetes, and physical activity were adjusted.

b In this model, sex, smoking, alcohol consumption, ethnicity, education levels, married status, gross family income, history of diabetes, and physical activity were adjusted.

### The Contributions of all Exposure Variables to the Incidence of Hypertension

Supplementary Table S5 shows that TV or computer usage mostly contributed to the incidence of hypertension (*HR*: 1.411; *95% CI*: 1.307–1.524; and *p* < 0.001) when all physical exercise types were analyzed simultaneously. When dietary preferences were analyzed simultaneously, fast food was the most promising predictor of incident hypertension (*HR*: 1.263; *95% CI*: 1.140–1.398; and *p* < 0.001). When physical exercise types, dietary preferences, and obesity patterns were analyzed simultaneously, an obesity pattern of G+/A+ was the most promising predictor of incident hypertension (*HR*: 1.845; *95% CI*: 1.715–1.985; and *p* < 0.001).

## Discussion

This study was conducted to examine the associations of specific types of physical exercise, dietary preferences, and obesity patterns with the incidence of hypertension in the Chinese adult population. The results implied that martial arts, gymnastics, and ping pong were associated with a lower risk of hypertension. However, dietary preferences for fast food, soft/sugared drinks, and salty snack food, as well as TV or computer usage increased the risk of hypertension. Furthermore, compared to subjects with G-/A-, subjects with G+/A- or G-/A+ or G+/A+ had an increased risk of hypertension. Furthermore, an obesity pattern of G+/A+ was the most promising predictor of incident hypertension.

Physical exercise is essential to reduce blood pressure and is broadly recommended to control and treat hypertension by the current American and European hypertension guidelines [19–21]. However, little was known on the associations of specific types of physical exercise with the risk of hypertension. A previous study reported that subjects participating in swimming, basketball, and aerobics had decreased all-cause and cardiovascular mortality [22]. In this study, subjects preferring martial arts, gymnastics, and ping pong had a lower risk of hypertension, which was consistent with previous studies [23, 24]. It was suggested that physical exercise could improve the redox state and functional and biochemical properties of the cardiovascular system [25, 26]. Furthermore, physical exercise could modulate superoxide dismutase activity and improve endothelial function via inhibiting oxidative stress and inflammatory markers [27, 28]. As is well known, regular physical exercise improves energy metabolism and reduces adiposity, which is positively associated with hypertension [29]. Therefore, physical exercise was considered beneficial for hypertension and cardiovascular disease.

In this study, TV or computer usage was associated with a higher risk of hypertension, which was consistent with previous studies [30, 31]. TV or computer usage, as sedentary behavior, was associated with consumption of unhealthy foods, overeating, tobacco smoking, and physical inactivity [32–34]. These risk factors linked TV or computer usage to the risk of hypertension. Furthermore, TV or computer usage could reduce muscle contraction and increase vascular inflammation, which would elevate blood pressure [35, 36].

Subjects preferring fast food, soft/sugared drinks, and salty snack food had a higher risk of hypertension in this study. Fast food was a feature of western dietary patterns and related to a higher risk of hypertension, which was consistent with the findings of this study [37]. In recent decades, fast-food consumption has been growing rapidly in China [38]. As is well known, fast food is rich in unhealthy fats, salt, and sugar, which promoted the development of obesity and hypertension [39]. Therefore, it was feasible that fast food preference was associated with a higher risk of hypertension. On the other hand, soft/sugared drinks were linked to an increase in serum triglycerides levels, caloric intake, and insulin resistance, which are strongly associated with obesity and hypertension [40, 41]. However, dietary preference for vegetables was not associated with the development of hypertension in this study. The possible reasons were as follows: Firstly, the proportion of subjects preferring vegetables was very high in both normal and hypertensive subjects. Thus, the protective effect of vegetables might be masked. Secondly, this study focused on the dietary habit of vegetable consumption rather than on the specific types of vegetables. Therefore, vegetables unrelated to hypertension might confuse the actual effect on hypertension of vegetables as a whole.

Obesity patterns were used to establish how the co-existence of general and abdominal obesity affected the risk of hypertension. The results suggested that subjects with at least one of general or abdominal obesity had an increased incidence of hypertension. Furthermore, subjects with both general and abdominal obesity had a higher risk of hypertension than those with only one of general and abdominal obesity, which was similar to a previous study [42]. BMI and WC were considered as independent risk factors for hypertension and should be together used to predict hypertension. Therefore, the findings of this study provided further evidence on the co-associations of general and abdominal obesity with the risk of hypertension.

This study found that there were significant interactions between sex and physical exercise and dietary preferences on hypertension. A previous study reported that men were more likely to develop hypertension than women among young adults [43, 44]. Furthermore, it was documented that sex affected motivation toward physical exercise and modified the association of physical exercises with health outcomes [45, 46]. On the other hand, compared to men, women were more likely to consume vegetables but less likely to consume soft drinks [47]. Therefore, that mentioned above supports the finding of this study.

### Strengths and Limitations

This study has the advantage of a large-scale representative sample of the Chinese population. Therefore, the results of this study were convictive. The population in this study included a geographically diverse population, which made the results generalizable to the greater Chinese population. Furthermore, the associations of obesity patterns and specific types of physical exercise with the risk of hypertension were rarely investigated, especially in the Chinese population. Therefore, this study was expected to provide additional evidence and insights for the prevention of hypertension. However, there were also some limitations in this study. Firstly, since the salt intake of participants in the CHNS was unavailable, the dietary-related information failed to be adjusted. Secondly, the population analyzed in this study is limited to the ethnically Chinese. Researchers should be cautious in extrapolating the conclusion to other ethnicities. Thirdly, the data on physical exercise and dietary preferences were collected using a self-reported questionnaire. There might be measurement bias. Fourthly, since dietary intakes were not available in the CHNS, dietary preferences but not dietary intake were used to evaluate the association of diet with the risk of hypertension.

### Conclusion

Martial arts, gymnastics, and ping pong were associated with a lower risk of hypertension. Dietary preferences of fast food, soft/sugared drinks, and salty snack food, as well as TV or computer usage, increased the risk of hypertension. Obese subjects were more likely to develop hypertension. Furthermore, the obesity pattern of both general and abdominal obesity was the most promising predictor of incident hypertension. The findings of this study have important public health implications regarding unhealthy dietary preferences and which type of physical exercise benefited cardiovascular health.

## Data Availability

The datasets analyzed during the current study are available on the CHNS website: http://www.cpc.unc.edu/projects/china.

## Supplementary Material

Supplementary Figure S1 The Kaplan-Meier curves of specific types of physical exercise with the incidence of hypertension (Lifestyle, obesity, and hypertension, China, 2021). A: Martial arts, B: Track and field, C: Gymnastics, D: Soccer or basketball, E: Badminton or volleyball, F: Ping pong, G: Reading or writing, and H: TV or computer usage. The x-axis represented age-scale, which was calculated as the time from birth year to the occurrence of hypertension, death, loss to follow-up, or the end of this study, whichever came first.

Supplementary Figure S2 The Kaplan-Meier curves of obesity patterns and dietary preferences with the incidence of hypertension (Lifestyle, obesity, and hypertension, China, 2021). A: Obesity patterns, B: Fast food, C: Soft/sugared drinks, D: Salty snack food, E: Vegetables, and F: Fruits. The x-axis represented age-scale, which was calculated as the time from birth year to the occurrence of hypertension, death, loss to follow-up, or the end of this study, whichever came first.
