# Supplementary figures and images for: Specific Types of Physical Exercises, Dietary Preferences, and Obesity Patterns With the Incidence of Hypertension: A 26-years Cohort Study
Source: Int J Public Health. 2022 Jan 27;66:1604441. doi: 10.3389/ijph.2021.1604441 (PMC8830503; doi:10.3389/ijph.2021.1604441)

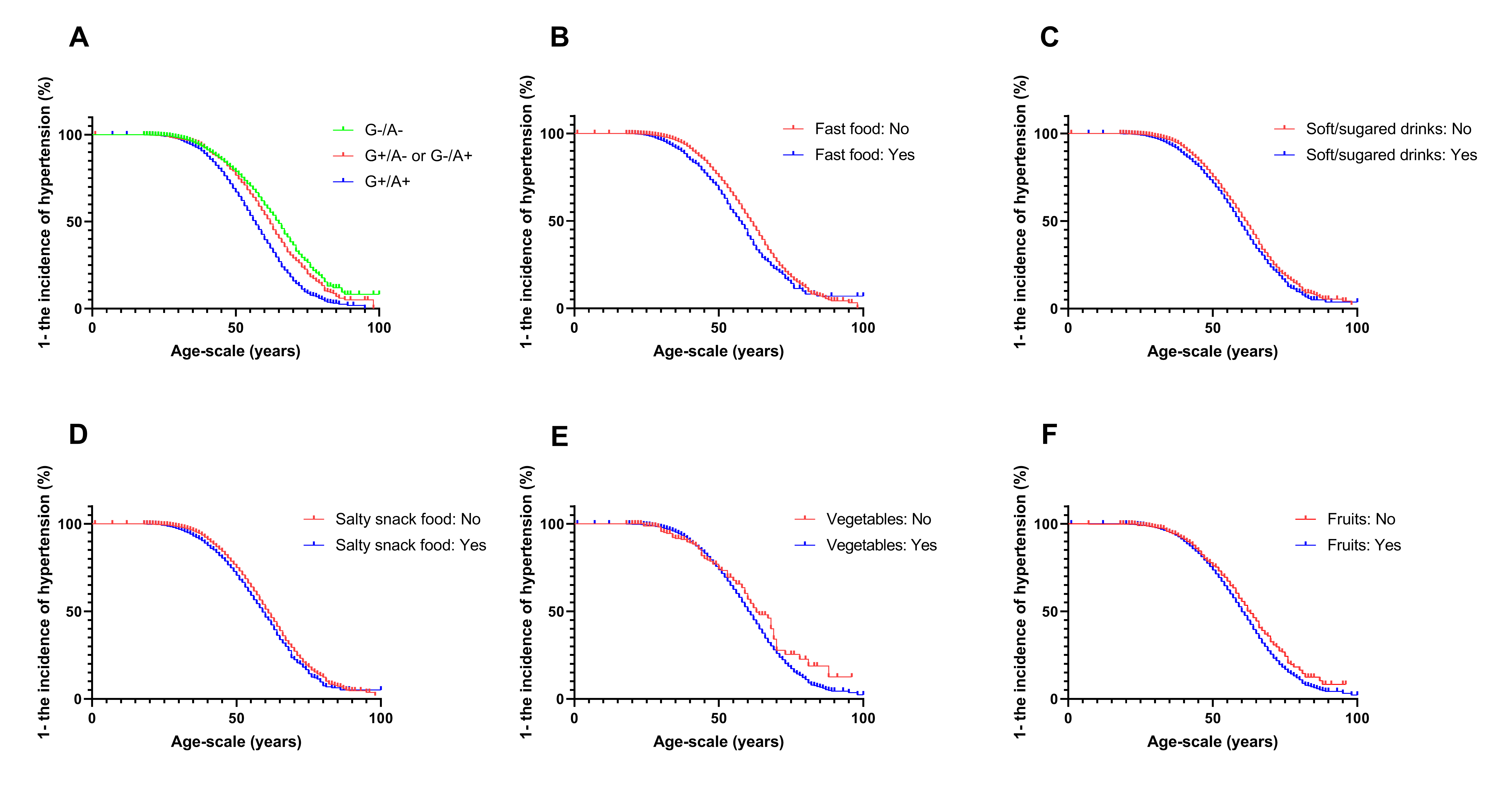

Supplement: Supplementary file 1 [file Image2.TIF]

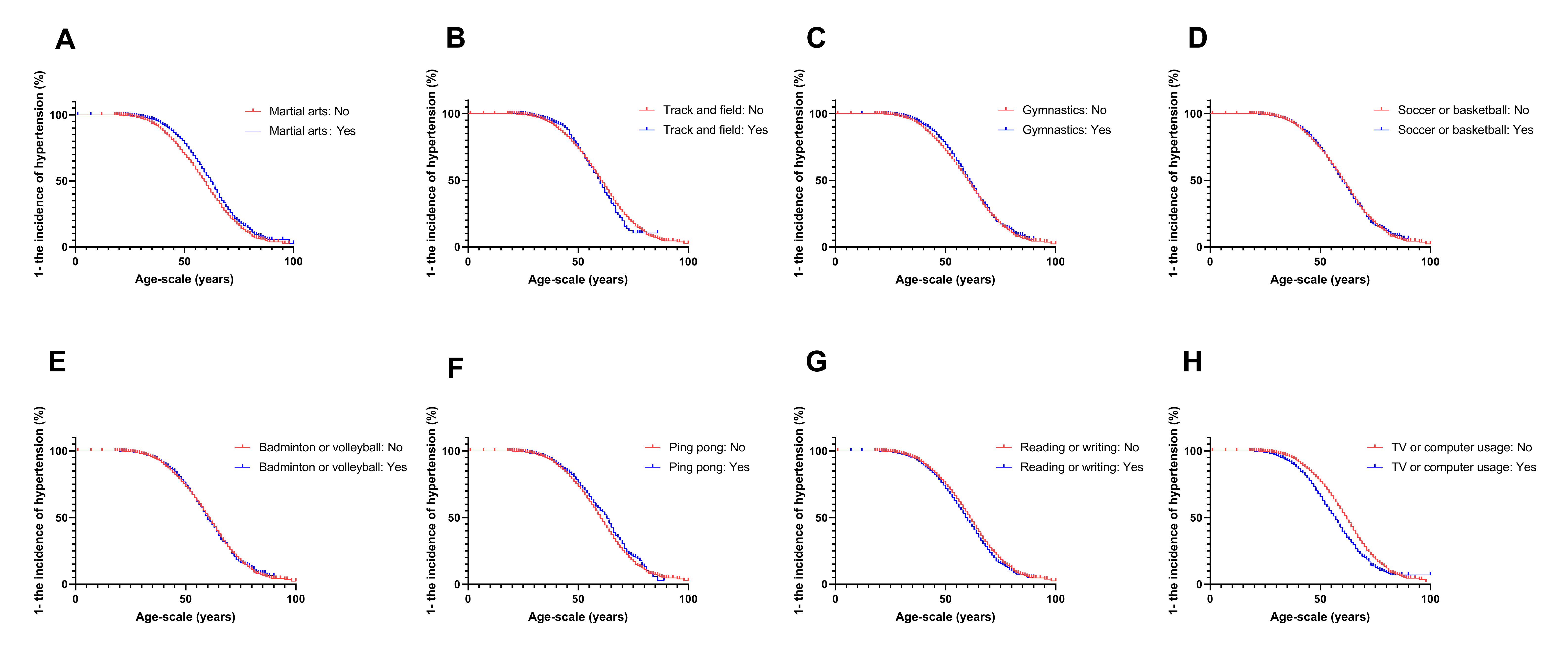

Supplement: Supplementary file 2 [file Image1.TIF]
