# Supplementary material for: Specific Types of Physical Exercises, Dietary Preferences, and Obesity Patterns With the Incidence of Hypertension: A 26-years Cohort Study
Source: Int J Public Health. 2022 Jan 27;66:1604441. doi: 10.3389/ijph.2021.1604441 (PMC8830503; doi:10.3389/ijph.2021.1604441)
Supplement: Supplementary file 3 [file DataSheet1.docx]

Table S1 The correlations between physical exercise types using Phi coefficient

| Physical exercise types | Martial arts | Track and field | Gymnastics | Soccer or basketball | Badminton or volleyball | Ping pong | Reading or writing | TV or computer usage |
| --- | --- | --- | --- | --- | --- | --- | --- | --- |
| Martial arts | 1.000 | 0.159 | 0.543 | 0.499 | 0.494 | 0.128 | 0.141 | 0.092 |
| Track and field | - | 1.000 | 0.192 | 0.195 | 0.196 | 0.113 | 0.151 | 0.087 |
| Gymnastics | - | - | 1.000 | 0.662 | 0.658 | 0.113 | 0.156 | 0.235 |
| Soccer or basketball | - | - | - | 1.000 | 0.982 | 0.139 | 0.161 | 0.291 |
| Badminton or volleyball | - | - | - | - | 1.000 | 0.144 | 0.166 | 0.294 |
| Ping pong | - | - | - | - | - | 1.000 | 0.167 | 0.068 |
| Reading or writing | - | - | - | - | - | - | 1.000 | 0.220 |
| TV or computer usage | - | - | - | - | - | - | - | 1.000 |

Table S2 The correlations between food preferences using Phi coefficient

| Food preferences | Fast food | Soft/sugared drinks | Salty snack food | Vegetables | Fruits |
| --- | --- | --- | --- | --- | --- |
| Fast food | 1.000 | 0.351 | 0.583 | 0.013 | 0.081 |
| Soft/sugared drinks | - | 1.000 | 0.370 | 0.054 | 0.106 |
| Salty snack food | - | - | 1.000 | 0.044 | 0.143 |
| Vegetables | - | - | - | 1.000 | 0.336 |
| Fruits | - | - | - | - | 1.000 |

Table S3 The full models of the associations of specific type of physical exercises with the incidence of hypertension (*HR* (*95% CI*))

| Factors | Model 1 | Model 2 | Model 3 | Model 4 | Model 5 | Model 6 | Model 7 | Model 8 |
| --- | --- | --- | --- | --- | --- | --- | --- | --- |
| Martial arts | 0.792(0.743,0.845) | - | - | - | - | - | - | - |
| Track and field | - | 0.947(0.831,1.079) | - | - | - | - | - | - |
| Gymnastics | - | - | 0.884(0.825,0.948) | - | - | - | - | - |
| Soccer or basketball | - | - | - | 0.974(0.902,1.051) | - | - | - | - |
| Badminton or volleyball | - | - | - | - | 0.970(0.898,1.047) | - | - | - |
| Ping pong | - | - | - | - | - | 0.855(0.767,0.953) | - | - |
| Reading or writing | - | - | - | - | - | - | 0.994(0.923,1.071) | - |
| TV or computer usage | - | - | - | - | - | - | - | 1.418(1.315,1.529) |
| Smoking | 1.041(0.955,1.134) | 1.043(0.957,1.136) | 1.044(0.958,1.137) | 1.044(0.958,1.137) | 1.044(0.958,1.137) | 1.041(0.956,1.134) | 1.043(0.958,1.136) | 1.023(0.940,1.115) |
| Alcohol consumption | 1.203(1.114,1.299) | 1.200(1.112,1.296) | 1.203(1.114,1.299) | 1.200(1.112,1.296) | 1.201(1.112,1.296) | 1.200(1.112,1.295) | 1.200(1.111,1.295) | 1.176(1.089,1.270) |
| Ethnicity | 0.810(0.710,0.923) | 0.828(0.726,0.944) | 0.827(0.725,0.942) | 0.828(0.726,0.944) | 0.828(0.726,0.944) | 0.830(0.729,0.946) | 0.829(0.727,0.945) | 0.807(0.706,0.921) |
| Sex | 1.023(0.939,1.115) | 1.024(0.940,1.115) | 1.027(0.943,1.119) | 1.025(0.941,1.116) | 1.025(0.941,1.116) | 1.021(0.937,1.112) | 1.024(0.940,1.116) | 1.007(0.924,1.097) |
| BMI | 1.077(1.067,1.087) | 1.075(1.065,1.085) | 1.076(1.066,1.086) | 1.075(1.065,1.085) | 1.075(1.065,1.085) | 1.076(1.065,1.086) | 1.075(1.065,1.085) | 1.075(1.064,1.086) |
| Education degree | 1.259(1.192,1.329) | 1.232(1.167,1.300) | 1.249(1.183,1.319) | 1.233(1.168,1.302) | 1.234(1.168,1.303) | 1.238(1.174,1.307) | 1.231(1.162,1.304) | 1.191(1.127,1.259) |
| Married status | 0.603(0.550,0.661) | 0.592(0.540,0.649) | 0.594(0.542,0.651) | 0.592(0.540,0.649) | 0.592(0.540,0.650) | 0.592(0.540,0.649) | 0.592(0.540,0.649) | 0.599(0.546,0.656) |
| Region | 1.262(1.183,1.347) | 1.302(1.221,1.388) | 1.288(1.208,1.374) | 1.300(1.219,1.386) | 1.300(1.219,1.386) | 1.287(1.207,1.372) | 1.302(1.219,1.390) | 1.309(1.228,1.396) |
| Gross family income | 1.002(0.931,1.078) | 0.966(0.899,1.038) | 0.978(0.910,1.052) | 0.968(0.900,1.040) | 0.968(0.901,1.040) | 0.965(0.898,1.037) | 0.965(0.898,1.037) | 0.965(0.898,1.037) |
| History of diabetes | 1.074(0.932,1.237) | 1.032(0.895,1.190) | 1.040(0.902,1.200) | 1.033(0.896,1.190) | 1.033(0.896,1.191) | 1.037(0.899,1.196) | 1.032(0.896,1.190) | 1.088(0.941,1.258) |

Model 1: Martial arts was independent variable.

Model 2: Track and field was independent variable.

Model 3: Gymnastics was independent variable.

Model 4: Soccer or basketball was independent variable.

Model 5: Badminton or volleyball was independent variable.

Model 6: Ping pong was independent variable.

Model 7: Reading or writing was independent variable.

Model 8: TV or computer usage was independent variable.

Table S4 The full models of the associations of obesity patterns and dietary preferences with the incidence of hypertension (*HR* (*95% CI*))

| Factors | Model 1 | Model 2 | Model 3 | Model 4 | Model 5 | Model 6 |
| --- | --- | --- | --- | --- | --- | --- |
| G+/A- or G-/A+ | 1.205(1.108,1.311) | - | - | - | - | - |
| G+/A+ | 1.747(1.626,1.876) | - | - | - | - | - |
| Fast food | - | 1.381(1.269,1.504) | - | - | - | - |
| Soft/sugared drinks | - | - | 1.233(1.157,1.314) | - | - | - |
| Salty snack food | - | - | - | 1.225(1.139,1.316) | - | - |
| Vegetables | - | - | - | - | 1.194(0.951,1.498) | - |
| Fruits | - | - | - | - | - | 1.155(1.021,1.307) |
| Smoking | 1.028(0.945,1.119) | 1.026(0.942,1.118) | 1.031(0.946,1.122) | 1.026(0.942,1.117) | 1.035(0.950,1.128) | 1.040(0.955,1.133) |
| Alcohol consumption | 1.208(1.119,1.303) | 1.200(1.111,1.295) | 1.203(1.114,1.298) | 1.202(1.113,1.297) | 1.203(1.115,1.299) | 1.204(1.115,1.300) |
| Ethnicity | 0.835(0.735,0.948) | 0.817(0.717,0.932) | 0.807(0.708,0.920) | 0.816(0.716,0.930) | 0.825(0.724,0.941) | 0.822(0.721,0.937) |
| Sex | 1.014(0.932,1.104) | 1.003(0.920,1.093) | 1.006(0.924,1.096) | 1.001(0.919,1.090) | 1.021(0.937,1.112) | 1.019(0.936,1.110) |
| Physical activity | 0.843(0.777,0.916) | 0.840(0.772,0.913) | 0.847(0.780,0.921) | 0.844(0.776,0.917) | 0.844(0.776,0.917) | 0.844(0.777,0.917) |
| BMI | - | 1.076(1.065,1.086) | 1.076(1.066,1.087) | 1.076(1.066,1.087) | 1.075(1.065,1.086) | 1.075(1.065,1.086) |
| Education degree | 1.252(1.186,1.321) | 1.241(1.175,1.310) | 1.255(1.188,1.325) | 1.250(1.183,1.320) | 1.253(1.187,1.323) | 1.252(1.185,1.322) |
| Married status | 0.574(0.524,0.630) | 0.598(0.546,0.656) | 0.598(0.546,0.656) | 0.596(0.543,0.653) | 0.591(0.539,0.648) | 0.593(0.541,0.651) |
| Region | 1.297(1.217,1.382) | 1.291(1.211,1.377) | 1.280(1.201,1.365) | 1.283(1.204,1.368) | 1.285(1.205,1.370) | 1.283(1.203,1.367) |
| Gross family income | 0.977(0.910,1.049) | 0.975(0.907,1.047) | 0.978(0.910,1.051) | 0.968(0.901,1.040) | 0.972(0.904,1.044) | 0.971(0.904,1.043) |
| History of diabetes | 1.053(0.917,1.210) | 1.057(0.917,1.219) | 1.080(0.937,1.246) | 1.051(0.911,1.213) | 1.038(0.899,1.198) | 1.041(0.902,1.202) |

Model 1: Obesity patterns were independent variable.

Model 2: Fast food was independent variable.

Model 3: Soft/sugared drinks was independent variable.

Model 4: Salty snack food was independent variable.

Model 5: Vegetables was independent variable.

Model 6: Fruits was independent variable.

Table S5 The contributions of all exposure variables to the incidence of hypertension

| Exposure variables | Model 1^※^ | | | Model 2^＃^ | | | Model 3^§^ | | |
| --- | --- | --- | --- | --- | --- | --- | --- | --- | --- |
|  | *HR* | *95% CI* | *P* | *HR* | *95% CI* | *P* | *HR* | *95% CI* | *P* |
| Martial arts | 0.813 | 0.754-0.877 | < 0.001 | - | - | - | 0.818 | 0.759-0.882 | < 0.001 |
| Track and field | 0.980 | 0.856-1.122 | 0.768 | - | - | - | 0.963 | 0.839-1.104 | 0.587 |
| Gymnastics | 0.902 | 0.821-0.992 | 0.033 | - | - | - | 0.888 | 0.808-0.977 | 0.015 |
| Soccer or basketball | 1.256 | 0.842-1.874 | 0.264 | - | - | - | 1.241 | 0.843-1.828 | 0.274 |
| Badminton or volleyball | 0.869 | 0.585-1.292 | 0.489 | - | - | - | 0.867 | 0.591-1.272 | 0.465 |
| Ping pong | 0.882 | 0.789-0.986 | 0.027 | - | - | - | 0.895 | 0.802-0.999 | 0.047 |
| Reading or writing | 0.987 | 0.915-1.065 | 0.739 | - | - | - | 0.979 | 0.908-1.056 | 0.586 |
| TV or computer usage | 1.411 | 1.307-1.524 | < 0.001 | - | - | - | 1.343 | 1.241-1.453 | < 0.001 |
| Fast food | - | - | - | 1.263 | 1.140-1.398 | < 0.001 | 1.212 | 1.093-1.343 | < 0.001 |
| Soft/sugared drinks | - | - | - | 1.148 | 1.073-1.229 | < 0.001 | 1.100 | 1.027-1.178 | 0.006 |
| Salty snack food | - | - | - | 1.050 | 0.963-1.144 | 0.270 | 1.015 | 0.931-1.107 | 0.732 |
| Vegetables | - | - | - | 1.099 | 0.866-1.393 | 0.438 | 1.097 | 0.859-1.400 | 0.459 |
| Fruits | - | - | - | 1.065 | 0.934-1.214 | 0.348 | 1.086 | 0.951-1.241 | 0.223 |
| G+/A- or G-/A+ | - | - | - | - | - | - | 1.268 | 1.164-1.381 | < 0.001 |
| G+/A+ | - | - | - | - | - | - | 1.845 | 1.715-1.985 | < 0.001 |

^※^In this model, sex, smoking, alcohol consumption, ethnicity, education levels, married status, regions, gross family income, history of diabetes, and BMI were adjusted.

^＃^In this model, sex, smoking, alcohol consumption, ethnicity, education levels, married status, regions, gross family income, history of diabetes, BMI, and physical activity were adjusted.

^§^In this model, sex, smoking, alcohol consumption, ethnicity, education levels, married status, regions, gross family income, and history of diabetes were adjusted.
